# Nomogram Predicting Parametrial Involvement Based on the Radical Hysterectomy Specimens in the Early-Stage Cervical Cancer

**DOI:** 10.3389/fsurg.2021.759026

**Published:** 2021-10-27

**Authors:** Chunbo Li, Shimin Yang, Keqin Hua

**Affiliations:** Department of Obstetrics and Gynecology, Obstetrics and Gynecology Hospital of Fudan University, Shanghai, China

**Keywords:** cervical cancer, nomogram, parametrial involvement, radical hysterectomy, decision curve analysis

## Abstract

**Objective:** Radical hysterectomy (RH) is the surgical standard for the treatment of the early-stage cervical cancer (CC). However, this procedure is associated with a high rate of adverse impact on the quality of the life of the patient. Since the rate of parametrial involvement (PI) is low for the patients with the early-stage CC, some authors believe that the patients with the early-stage CC may benefit from the less radical surgery. This study aims to estimate the incidence of the PI in the patients with the early-stage CC and establish a simple nomogram to identify a cohort of the patients with low risk of the PI who may benefit from the less radical surgery.

**Methods:** All the patients who underwent the RH and pelvic lymphadenectomy were included from 2013 to 2018. The significant independent predictors were identified through the Cox regression analysis and then incorporated into a nomogram to predicate the PI. The calibration plots and receiver operating characteristic (ROC) curves were used to assess the predictive accuracy of the nomogram.

**Results:** A total of 4,533 patients met the inclusion criteria and 441 women (9.7%) had the PI. The positive PI rate in the ≤2 cm group (1.2%) was significantly lower compared to >2– ≤4 cm (6.2%) or >4 cm (22.4%) groups. The multivariate analyses revealed that tumor size (*p* = 0.002), lymphovascular space invasion (LVSI) (*p* = 0.001), vaginal involvement (VI) (*p* < 0.001), status of the pelvic lymph nodes (PLNs) (*p* = 0.001), and depth of stromal invasion (DSI) (*p* < 0.001) were the independent prognostic factors of the PI. Finally, the five variables were combined to construct the nomogram model. The concordance indexes (C-indexes) of the PI were 0.756 (95% CI 0.726–0.786) for the internal validation and 0.729 (95% CI 0.678–0.780) for the external validation. The calibration plots further showed good consistency between the nomogram prediction and the actual observation.

**Conclusion:** This study confirmed that the patients with tumor size 2 cm or smaller were at very low risk for the PI. If other variables such as negative LVSI, DSI <50%, no VI, and negative PLN were limited, the risk would reduce significantly. Meanwhile, a simple nomogram based on the significant clinicopathological characteristics could be used as a tool for the clinicians to predict the PI among the patients with the early-stage CC, who might benefit from a less radical surgery.

## Introduction

Radical hysterectomy (RH) with pelvic lymphadenectomy has become the surgical standard for the treatment of the early-stage cervical cancer (CC) (1). The procedure requires en bloc resection of the uterus and cervix along with the surrounding parametria to remove any micrometastatic lesions that have spread from the cervix (2). However, parametrectomy is the most difficult step of the RH and is generally considered as the main source of substantial complications. The occurrence of the complications can be attributed to the parametrial resection as part of the RH (3). The parametrium is rich in the vasculature and autonomic nerve fibers and transsection of these tissues may result in the hemorrhage and devascularization of tissue leading to the formation of the fistulae or bladder denervation (4). Some surgeons advocate a “nerve-sparing” technique that may minimize the occurrence of these complications, but the technique requires a specific learning curve and high surgical skills before being generalized (5, 6).

In contrast to the RH, hysterectomy can preserve the parametrial tissue and is associated with a significant reduction in the incidence of operative morbidity (7). Recently, some studies have shown that there is no significant difference in the recurrence rate and overall survival rate among the patients with the early-stage CC undergoing the simple extrafascial hysterectomy and RH (8, 9). The theoretical basis is that for the patients with the early-stage CC, the possibility of infiltration of the parametrial tissue is low (10). Several studies have reported that women with small tumor (<2 cm), negative pelvic lymph nodes (PLNs), and no lymphovascular space invasion (LVSI) have low parametrial involvement (PI) <1% (11, 12). Therefore, some authors report that for women with the low-risk early-stage CC, the reduction in radicality does not seem to affect their prognosis. In a large retrospective study, Covens et al. investigated that the patients with a tumor size <20 mm, no LVSI, depth of stromal invasion (DSI) <10 mm, and negative PLN have a 0.6% probability of the PI and so they can benefit from the less radical surgery (12). In view of these findings, there has been great interest in considering the less radical resection with simple hysterectomy in women with the low-risk early-stage CC.

Despite the rationale for the conservative resection for the early-stage CC, the data are limited and based predominately on the small institutional series. In this study, the incidence of the PI in the women with the early-stage CC was estimated, who underwent the RH to evaluate the possible risk factors related to the spread of the parametrial tissue. Meanwhile, in an effort to estimate whether certain patients with the early-stage CC are suitable for less radical surgery, this study analyzed the identified risk factors to develop a nomogram to predict which women have a lower risk of the PI.

## Methods

After being approved by the Medical Ethics Committee of the hospital, the medical records of the patients diagnosed as the early-stage CC (IA1 with LVSI, IA2-IIA2) were reviewed from 2013 to 2018. Preoperative clinical staging was evaluated according to the 2018 International Federation of Gynecology and Obstetrics (FIGO) criteria. All the patients had no evidence of the metastatic node or the PI in the preoperative examination. Patients with a clinical or MRI PI were excluded. Patients were also excluded if they received the neoadjuvant therapy before the primary surgery or did not undergo any radical surgery because the PI was not accessible. Then, all the operations were performed according to the surgical classification of the Querleu and Morrow RH: staged IB1-IIA2 was type C and staged IA1 with LVSI or IA2 was type B. Lymphadenectomy consisted of the systematic PLN dissection. Data were collected regarding the pathological factors including tumor size, histology, vaginal invasion (VI), depth of invasion, LVSI, PI, and status of PLN. We could acquire a pathological staging according to the variables.

All the specimens were analyzed by experienced gynecological pathologists. At the time of the gross examination, the parametrial margins were identified and submitted for the microscopic evaluation. If the tumor appeared to extend into the parametrium, contiguous full-thickness sections of the tumor in the cervix and parametrium were submitted. Tumor size was defined by the maximum diameter. The assessment of LVSI was made by the microscopic evaluation of HE-stained slides. With respect to the cut margin of the vaginal canal, a tumor-free space >10 mm in length was defined as a tumor-free surgical margin. Parametrial lymph node metastasis was classified as a positive PLN.

The pathological variables such as tumor size, PI, the status of the PLNs, DSI, LVSI, infiltration into the vagina, and histological subtypes were recorded. Descriptive statistics were carried out to describe the baseline characteristics of the patients. Continuous variables with normal distribution were shown as mean (SD) and nonnormal continuous variables were presented as median [interquartile range (IQR)]. Categorical variables were summarized in the terms of frequency and percentages. To construct and validate the nomogram, this study randomly divided all the patients into the training and validation cohorts in a ratio of 7:3. Univariable and multivariate Cox regression analysis were used to calculate the effect of the variable on the PI in the training group. By using these identified prognostic factors, this study constructed a nomogram for predicting the PI. The nomogram was validated internally in the training cohort and externally in the validation cohort. To evaluate the discriminative ability of the nomogram, this study used the concordance index (C-index) and the receiver operating characteristic curve (ROC) and assessed the area under the curve (AUC). The calibration curves were used to compare the association between the actual outcomes and the predicted probabilities. Both the discrimination and calibration were evaluated by using bootstrapping with 1,000 resamples. All the statistical analyses were performed by using the SPSS version 24.0 (SPSS Incorporation, Chicago, Illinois, USA) and the The R Foundation for Statistical Computing, Vienna, Austria (version 3.4.3; http://www.r-project.org/). A *p* < 0.05 was considered to be statistically significant.

## Results

**Figure 1:**
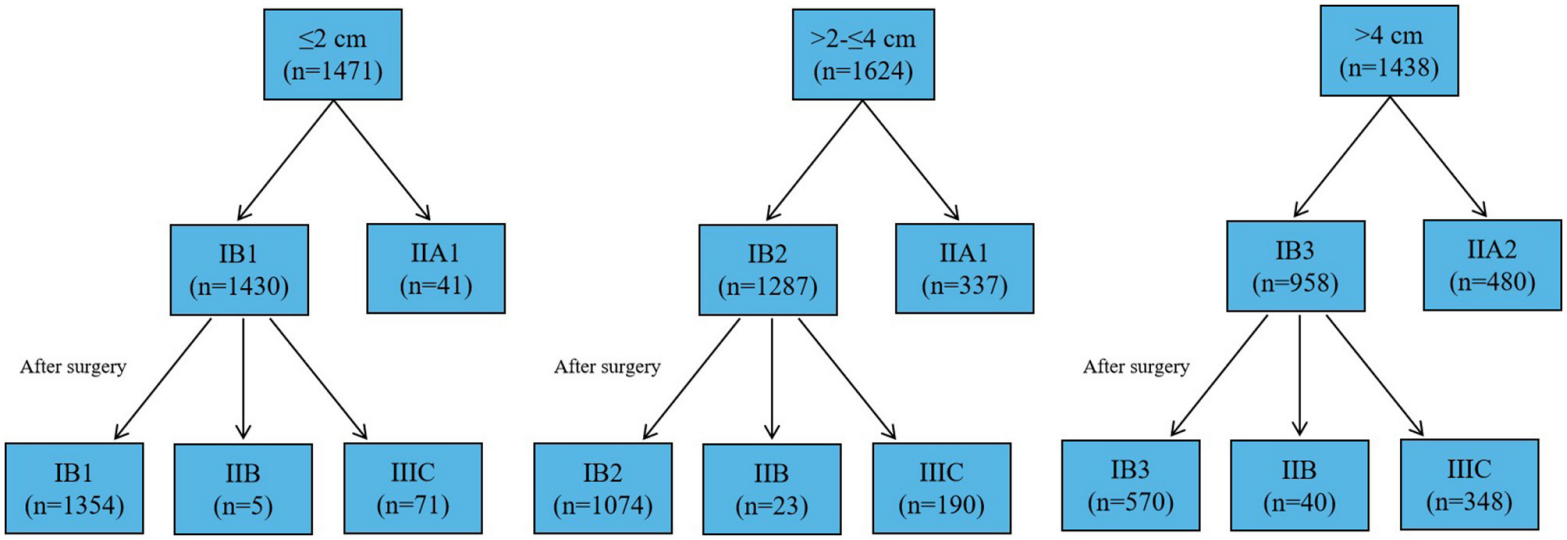
The progression of the stage after the surgery in all the patients.

A total of 4,533 women with the early-stage CC who underwent the RH met the criteria of this study. The patient characteristics and pathological factors for the entire cohort according to the size of tumor ≤2 cm, >2 and ≤4 cm, and >4 cm were summarized in Table 1. The average age of the patients ≤2 cm, >2 cm and ≤4 cm, and >4 cm was 46.7 ± 9.4 years, 48.9 ± 9.8 years, and 49.2 ± 9.7 years, respectively. Among 1,471 women with tumor size ≤2 cm, 78 women were positive PLN (5.3%), 105 women were vaginal involvement (7.1%), 299 women were positive LVSI (20.3%), and 194 women were DSI ≥50% (13.2%). Approximately, 76 (5.3%) patients with stage IB1 presented with higher staging according to the pathological samples (Figure 1). For women with a tumor size 2–4 cm, positive PLN was 17.7%, vaginal involvement was 20.8%, positive LVSI was 54.6%, and DSI ≥ 50% was 65.3%. Approximately, 213 (16.6%) patients with stage IB2 was high staging after surgery. For women with tumor size >4 cm, the rate of positive PLN, VI, LVSI, and DSI ≥50% increased significantly. The number of patients with high stage after surgery was 388 (40.5%).

**Table 1:** Clinical and pathological characteristics of the patients related to the size of the tumor.

	**Tumor size ≤ 2 cm (1,471)**	**Tumor size >2 and ≤4 cm (1,624)**	**Tumor size >4 cm (1,438)**	***P***
Age (years)	46.7 ± 9.4	48.9 ± 9.8	49.2 ± 9.7	0.000
Menopausal state				0.000
Yes	497	696	612	
No	974	928	826	
BMI (kg/m2)	21.7 ± 3.3	22.3 ± 3.5	22.1 ± 3.6	0.000
FIGO				0.000
≤ IB1	1366	0	0	
IB2	0	1287	0	
IB3	0	0	958	
IIA1	105	337	0	
IIA2	0	0	487	
Parametrial involvement				0.000
Positive	18 (1.2%)	101 (6.2%)	322 (22.4%)	
Negative	1453 (98.8%)	1523 (93.8%)	1116 (77.6%)	
Depth of stromal invasive				0.000
≥1/2	194 (13.2%)	1061 (65.3%)	1292 (89.8%)	
<1/2	1277 (86.8%)	563 (34.7%)	146 (10.2%)	
Infiltration into vagina				0.000
Positive	105 (7.1%)	337 (20.8%)	480 (33.4%)	
Negative	1366 (92.9%)	1287 (79.2%)	958 (66.6%)	
Surgical vaginal margin				0.000
Positive	41 (2.8%)	136 (8.4%)	180 (12.5%)	
Negative	1430 (97.2%)	1488 (91.6%)	1258 (87.5%)	
Status of pelvic lymph nodes				0.000
Positive	78 (5.3%)	288 (17.7%)	593 (41.2%)	
Negative	1393 (94.7%)	1336 (82.3%)	845 (58.8%)	
Histological subtype				0.000
Squamous cell cancer	1115 (75.8%)	1349 (83.1%)	1213 (84.4%)	
Adenocarcinoma	268 (18.2%)	180 (11.1%)	157 (10.9%)	
Adenosequamous cancer	76 (5.2%)	75 (4.6%)	56 (3.9%)	
Other subtype	12 (0.8%)	20 (1.2%)	12 (0.8%)	
Infiltration into uterine body				0.000
Positive	39 (2.7%)	219 (13.5%)	474 (33%)	
Negative	1432 (97.3%)	1405 (86.5%)	964 (67%)	
Lymph vascular space invasion				0.000
Positive	299 (20.3%)	887 (64.6%)	917 (63.8%)	
Negative	1172 (79.7%)	737 (45.4%)	521 (36.2%)	
Recurrence				0.000
Positive	29 (2.0%)	101 (6.2%)	199 (13.8%)	
Negative	1433 (97.4%)	1504 (92.6%)	1194 (83.0%)	
Unknown	9 (0.6%)	19 (1.2%)	45 (3.2%)	
Survival state				0.000
Death	28 (1.9%)	105 (6.5%)	233 (16.2%)	
Live	1443 (98.1%)	1519 (93.5%)	1205 (83.8%)	
Unknown	0 (0%)	0 (0%)	0 (0%)	

In order to further determine the incidence of the PI based on the risk factors, this study calculated the incidence of the PI by adding the limiting conditions (Table 2). For the patients with the PI, the incidence of the PI with tumor size ≤2 cm was 1.2% (18/1,471), which was significantly low compared to the patients with the tumor size >2 cm (*p* < 0.05). For the patients with the tumor size ≤2 cm, if VI was excluded, the risk of the PI would be reduced by half (0.61%). After excluding the positive PLN, only five patients had the PI (0.34%). Before surgery, the preoperative colposcopy and intraoperative biopsy could accurately confirm the diagnosis of the vaginal involvement and positive PLN, respectively. Given that the patients with the tumor size ≤2 cm have a lower risk of the PI, if the patients with the positive PLN or vaginal involvement are excluded, they may benefit from a less RH (Querleu and Morrow type A or type B). In addition, because the positive PLN and vaginal involvement represent the advanced stage, the patients may benefit from direct chemoradiotherapy. Since the presence or absence of LVSI had the lower risk of the PI for the patients with tumor size ≤2 cm compared to the tumor size >2 cm, it is less important to evaluate LVSI before surgery for the patients with the tumor size ≤2 cm. However, for the patients with a tumor size 2–4 cm, the risk of the PI is significantly increased. If all the risk factors were excluded, including the positive PLN, the presence of LVSI, and the vaginal involvement, only eight of 1,624 patients had the PI (0.49%). Therefore, some strictly selected patients could benefit from a less radical hysterectomy.

**Table 2:** The number of the total patients and those with parametrial invasion based on the tumor size, vaginal involvement, status of the pelvic lymph nodes, and the lymphovascular space invasion.

	**Tumor size ≤ 2 cm** **(***n*** = 1,471)**		**Tumor size >2 and ≤4 cm** **(***n*** = 1,624)**		**Tumor size > 4 cm** **(***n*** = 1,438)**	
	**PI (+)** **(***n =*** 18)**	**PI (–)** **(***n =*** 1453)**	**PI (+)** **(***n =*** 101)**	**PI (–)** **(***n =*** 1523)**	**PI (+)** **(***n =*** 322)**	**PI (–)** **(***n =*** 1116)**
VI (–)	9 (0.6%)	1,462 (99.4%)	51 (3.1%)	1,573 (96.9%)	144 (10.0%)	1,294 (90%)
LVSI (–)	3 (0.2%)	1,468 (99.8%)	21 (1.3%)	1,603 (98.7%)	52 (3.6%)	1,386 (96.4%)
PLN (–)	5 (0.3%)	1,466 (99.7%)	37 (2.3%)	1,587 (97.7%)	78 (5.4%)	1,360 (94.6%)
VI (–) and LVSI (–)	3 (0.2%)	1,468 (99.8%)	11 (0.7%)	1,613 (99.3%)	26 (1.8%)	1,412(98.2%)
PLN (–) and LVSI (–)	2 (0.1%)	1,469 (99.9%)	12 (0.7%)	1,612 (99.3%)	23 (1.6%)	1,415 (98.4%)
VI (–) and PLN (–)	5 (0.3%)	1,466 (99.7%)	23 (1.4%)	1,601 (98.6%)	40 (2.8%)	1,398 (97.2%)
VI (–), LVSI (–) and PLN (–)	2 (0.1%)	1,469 (99.9%)	8 (0.5%)	1,616 (99.5%)	12 (0.8%)	1,426 (99.2%)

*VI, Vaginal involvement; LVSI, lymph vascular space invasion; PLN, pelvic lymph nodes*.

**Figure 2:**
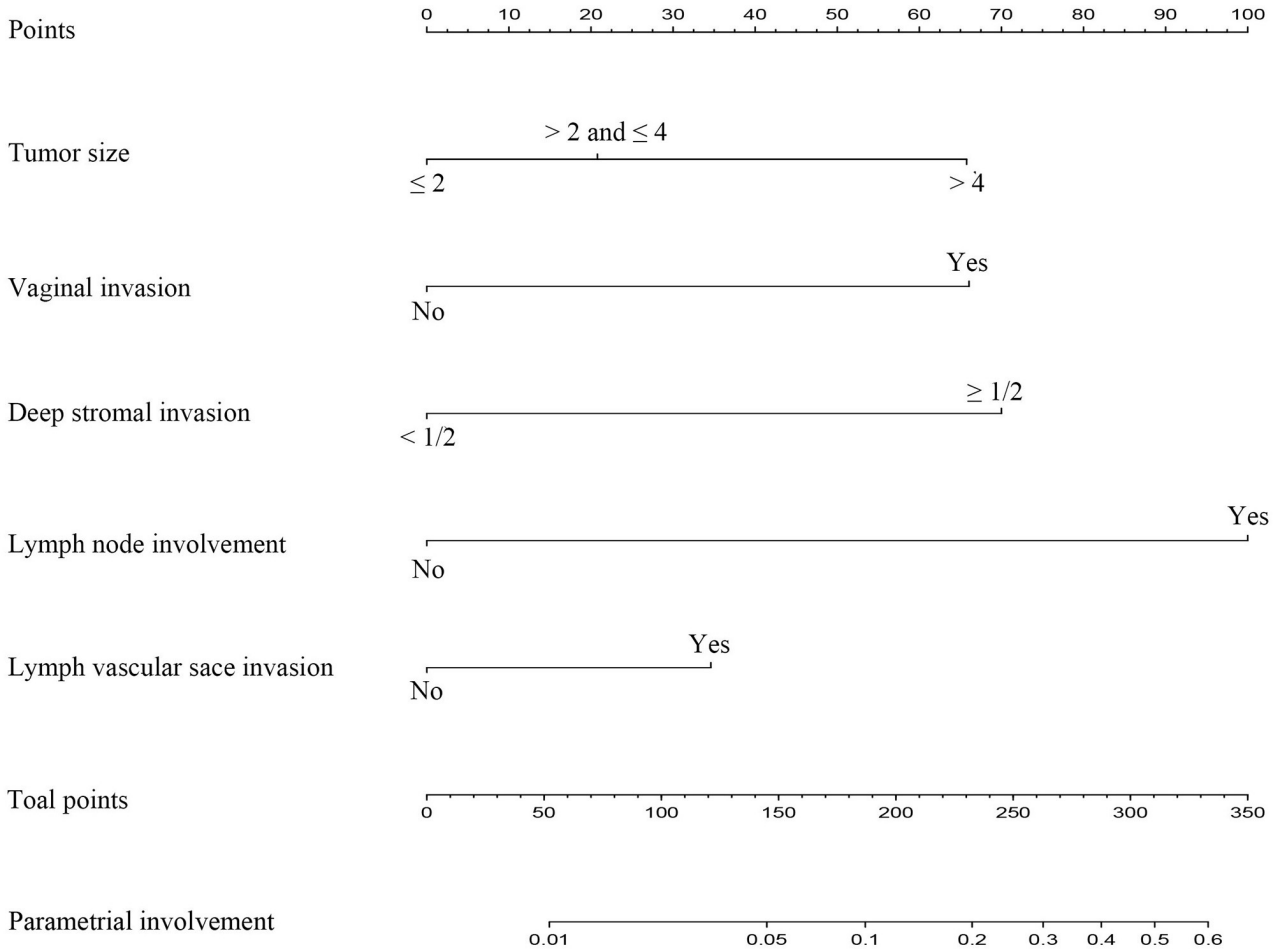
Nomogram for predicting the parametrial invasion of the patients with the early-stage cervical cancer with five available factors including tumor size, depth of the stromal invasion, vaginal involvement, status of the pelvic lymph nodes, and the lymphovascular space invasion.

**Figure 3:**
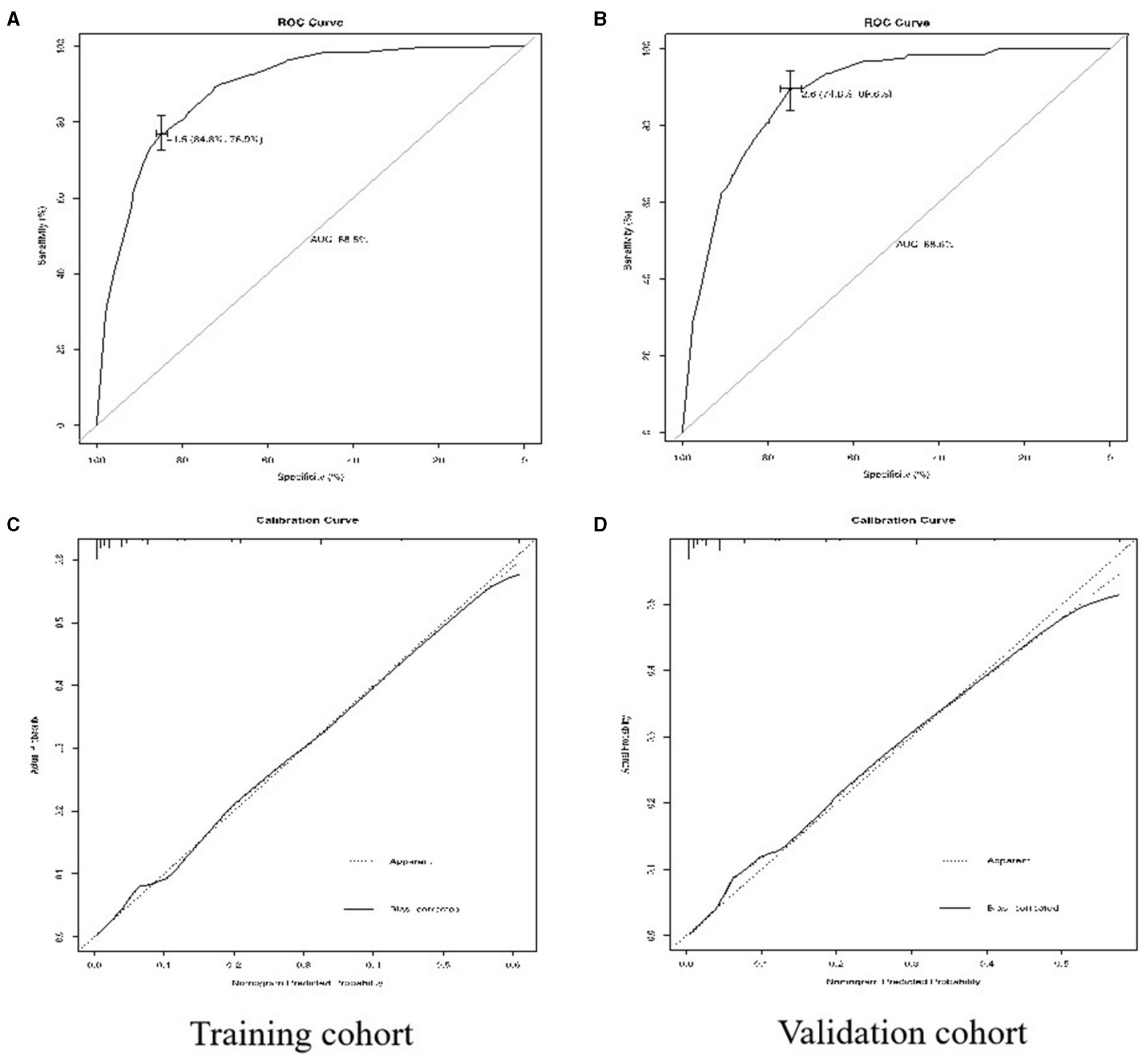
Receiver operating characteristic (ROC) curves for predicting the parametrial involvement (PI) **(A,B)** and the calibration plots **(C,D)** in the training cohorts and the validation cohorts.

**Figure 4:**
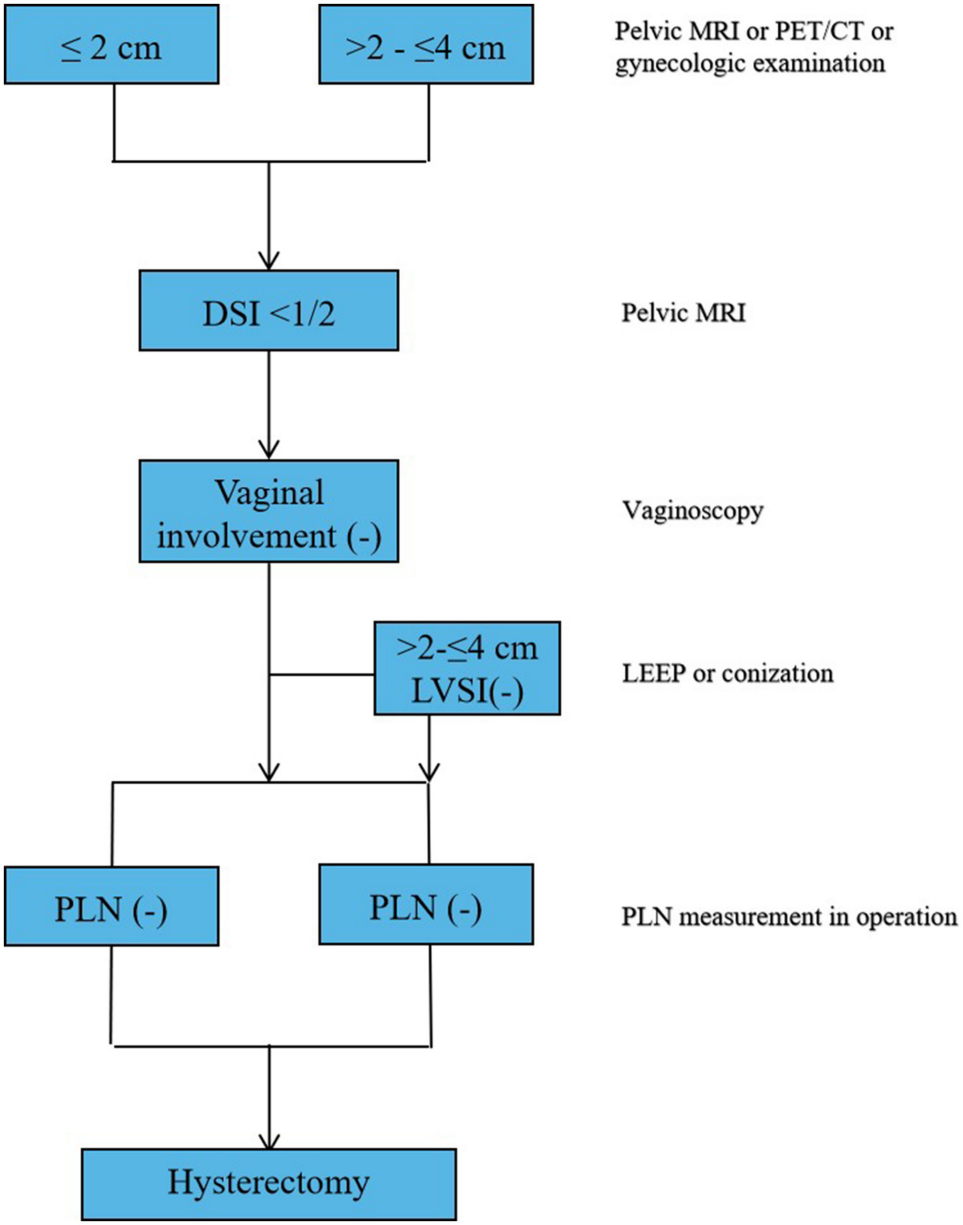
Summary of the main outcomes in this study.

Due to the more limitations of a single variable, this study established a simple nomogram to predict the PI in the early-stage CC and define the low-risk population who would benefit from a less RH (Table 3). According to the Cox regression analysis, the PI was strongly associated with tumor size (*p* = 0.002), the presence of LVSI (*p* < 0.001), vaginal involvement (*p* < 0.001), DSI ≥ 50% (*p* < 0.001), and positive PLN (*p* < 0.001) (Table 4). Taking into account the results of the multivariate Cox regression analysis and the clinical practice of CC, all the significant variables were used to create a nomogram for the PI. The prognostic nomogram for the PI was shown in Figure 2. By summing the scores associated with each variable and projecting the total scores to the bottom scale, the probability of the PI can be estimated. In this nomogram, PLN contributed the greatest impact on the PI, while LVSI had less impact on the PI. The C-index values and ROC curve are ordinarily used to evaluate the discriminatory power of the nomogram. In the training cohort, the C-index value for predicting the PI was 0.88 (95% CI 0.87–0.90) (Figure 3A). Similar results were found in the validation cohort for predicting the PI that was 0.88 (95% CI 0.87–0.90) (Figure 3B). This similarity of the results indicated that the model established by the nomogram was accurate. Moreover, this study calibrated the PI nomograms of the training and validation cohort and they were very close to the ideal curve (Figures 3C,D). This showed good consistency between the predictions of the nomogram and the actual observed outcomes in the two cohorts (Figure 4).

**Table 3:** Demographics and pathological characteristics of the patients in the training cohort and the validation cohort.

	**Train data**			**Test data**		
	**PI (+)**	**PI (–)**	***P***	**PI (+)**	**PI (–)**	***P***
	***n*** **= 316**	***n*** **= 2,864**		***n*** **= 125**	***n*** **= 1,228**	
Age (year)			0.178			0.386
<40	54	536		18	226	
40–60	208	1,945		86	836	
≥ 60	54	383		21	166	
Menopausal state			0.957			0.052
Yes	131	1,191		61	489	
No	185	1,671		64	739	
BMI (kg/m2)	22.5 ± 3.5	22.4 ± 3.8	0.825	21.9 ± 3.4	22.1 ± 3.3	0.623
FIGO			0.000			0.000
≤ IB1	6	928		3	429	
IB2	37	870		14	366	
IB3	94	582		50	232	
IIA1	44	262		15	121	
IIA2	135	222		43	80	
Tumor size			0.000			0.000
≤ 2cm	13	991		5	464	
>2 and ≤ 4 cm	74	1,069		27	454	
>4cm	229	804		93	312	
Depth of stromal invasive			0.000			0.000
≥ 1/2	302	1,503		121	621	
<1/2	14	1,361		4	607	
Infiltration into vagina			0.000			0.000
Positive	179	484		58	201	
Negative	137	2380		47	1,027	
Surgical vaginal margin			0.000			0.000
Positive	80	192		23	62	
Negative	236	2,672		102	1,166	
Status of pelvic lymph nodes			0.000			0.000
Positive	229	438		92	200	
Negative	87	2,426		33	1,028	
Histological subtype			0.140			0.205
Squamous cell cancer	270	2,303		108	996	
Adenocarcinoma	30	391		11	155	
Adenosequamous cancer	11	131		3	62	
Other subtypes	5	39		3	15	
Infiltration into uterine body			0.000			0.000
Positive	135	383		57	157	
Negative	181	2,481		68	1,071	
Lymph vascular space invasion			0.000			0.000
Positive	260	1,237		105	501	
Negative	56	1,627		20	727	

**Table 4:** The univariable and multivariable Cox regression model analyses of the parametrial invasion in the training cohort.

	**Univariable analysis**		**Multivariable analysis**	
	**OR (95%CI)**	***P***	**OR (95%CI)**	***P***
**Age (year)**				
<40	Reference		NR	
≥40 <60	1.061 (0.775–1.453)	0.710		
≥60	1.399 (0.939–2.086)	0.099		
**Histological subtype**				
Squamous cell cancer	Reference		Reference	
Adenocarcinoma	0.654 (0.442–0.969)	0.034	0.859 (0.534–1.380)	0.859
Adenosequamous cancer	0.716 (0.382–1.342)	0.298	0.947 (0.468–1.967)	0.947
Other subtype	1.094 (0.427–2.798)	0.852	1.005 (0.328–3.078)	0.993
**Surgical vaginal margin**				
Negative	Reference		Reference	
Positive	4.718 (3.520–6.322)	<0.001	1.288 (0.875–1.896)	0.199
**Depth of stromal invasive**				
<1/2	Reference		Reference	
≥1/2	19.533 (11.374–33.547)	<0.001	3.128(1.675–5.842)	<0.001
**Tumor size**				
≤ 2cm	Reference		Reference	
>2 and ≤ 4 cm	5.277 (2.908–9.574)	<0.001	1.426 (0.722–2.818)	0.307
>4cm	21.712 (12.322–38.259)	<0.001	2.907 (1.489–5.678)	0.002
**Infiltration into vagina**				
Negative	Reference		Reference	
Positive	6.425 (5.039–8.192)	<0.001	2.926 (2.146–3.990)	<0.001
**Infiltration into uterine body**				
Negative	Reference		Reference	
Positive	4.832 (3.772–6.188)	<0.001	1.736 (1.289–2.338)	<0.001
**Lymph vascular space invasion**				
Negative	Reference		Reference	
Positive	6.107 (4.533–8.227)	<0.001	1.847 (1.305–2.612)	0.001
**Status of pelvic lymph nodes**				
Negative	Reference		Reference	
Positive	14.579 (11.163–19.041)	<0.001	5.504 (4.156–7.556)	0.001

## Discussion

This study found that 1.2% of women with the tumor size ≤2 cm had the PI who undergoing RH. After excluding the risk factors of the presence of LVSI, positive PLN, and VI, the risk of the PI was 0.14%. However, as the size of tumor increases (>2 cm), the risk of the PI would also increase significantly. In this study, tumor size, LVSI, DSI, VI, and the status of PLN were all related to the occurrence of the PI (Figure 4). Then, this study established a simple nomogram to predicate the PI in the patients with the early-stage CC. Based on the results of the multivariate Cox regression analysis, five important nomogram predictors had been confirmed and provide a more accurate assessment and higher clinical application value for the prediction of the PI in the patients with the early-stage CC.

Consistent with the previous studies, this study showed that five risk factors were associated with the PI. Frumovitz et al. reported that the women with the tumor size 2 cm or smaller and no LVSI had a very low risk for the PI (10). In a large retrospective study, Covens et al. stated that the patients with the tumor size < 20 mm, no LVSI, DSI < 10 mm, and negative PLN had a 0.6% probability of the PI (8). Kasamatsu et al. reported that the patients with the small tumor size (≤2 cm) have a lower risk for the pathological PI (2%) (11). Then, based on the risk factors, many authors tried to estimate the appropriate models to select the patients. Kong et al. established a nomogram based on the tumor volume, the disruption of the cervical stromal ring on MRI, serum squamous cell carcinoma (SCC) antigen level, and menopausal status to predict the PI in the early-stage CC (12). However, this model presented more limitations. First, the model relied on more specific MRI criteria and its reproducibility is poor in a nonspecialized center. Second, the use of SCC as a tumor marker in a population containing nonsquamous cell cancers may be controversial. Benoit et al. proposed a nomogram to predict the PI in the early-stage CC (13). In this study, sentinel lymph node, tumor size, DSI, and LVSI were included. They proved that the tool raised the individual probability of the PI. However, a notable limitation was the relatively small minority of the patients with the PI. Meanwhile, most variables in this study were not significant according to the multivariate Cox regression analysis, which may suffer from some bias.

Different from their nomogram model, this study found that VI played a key role in the occurrence of the PI, which was proved by the Cox regression analysis. A study by Coutant et al. reported that three of six patients with the PI and three of 31 patients without the PI had vaginal involvement (*p* = 0.04) (13). It is known that the occurrence of the PI is associated with the rate of tumor spread to the vaginal wall. This provides clues that this study can predict the PI through the diagnosis of VI. However, with respect to vaginal involvement, the available data are few in the literature, but, generally, ultrasonography and MRI have been found to have low accuracy. Sozzi et al. considered that examination under anesthesia might improve the identification of VI (14). In our opinion, colposcopy for the examination of the cervix and vagina by using the magnifying light microscope, colposcope, and acetic acid is very valuable for detecting the vaginal involvement. In addition, if they are suggestive of a minor lesion, multiple punch biopsies may be appropriate. Unlike other variables, such as PLN, LVSI, and DSI that mainly depended on the pathological examination after surgery, preoperative colposcopy could accurately acquire evidence of VI. Therefore, this study suggested that for the patients with the early-stage CC, who undergo the less radical surgery, colposcopy must be performed to rule out VI before surgery. DSI seems to be another attractive factor for the prognosis of the PI. However, there are currently no consensuses that should describe the critical value of DSI. Many studies used 10 mm of DSI as its cutoff level (15, 16). However, the authors disagree with this critical level because the cervical thickness of each patient can affect DSI (16, 17). Although some authors recommend using the ratio between DSI and cervical thickness, this is more reliable than the universal DSI cutoff value, even though more than one-third of the internal value is traditionally used to successfully assess the intermediate risk for the PI (18). Thus, many studies defined DSI as having stromal involvement of more than 50% (19). In this study, the patients with the PI had a stromal invasion of more than 50%, whereas for those patients without the PI, the number is low for the stromal invasion of <50%. Because of this, this study strongly recommends using DSI of more than 50% to predict the development of the PI in the patients with low risk. This study also found that for the patients undergoing conization or loop electrosurgical excision procedure (LEEP), it seems difficult to evaluate DSI based on the excised samples. Thus, this study suggested that DSI more than 50% in the preoperative MRI of the pelvis may be suitable for predicting the risk of the PI as a cutoff value.

It is found that the size of the tumor is an independent prognostic factor in determining the stage of CC. According to the previous studies, the prognosis of the patients with smaller tumor (≤2 cm) is significantly better compared to the patients with larger tumors (20). In addition, the patients with small tumor size were found to have a low risk for the pathological PI (<2%) (21). In fact, this study also found that regardless of the existence of LVSI, PLNs, DSI (>1/2), and vaginal involvement, the risk of the PI with tumor size <2 cm was low. Therefore, tumor size (=2 cm) can be selected as the criterion for selecting the candidates for the less RH (Querleu and Morrow type A or type B). Accurate diagnosis of tumor size is an important standard for tailoring management. Although tumor size could be assessed by the gynecological examination before surgery, it is a limitation for the tumors located in the endocervical region. Epstein et al. showed a higher accuracy for ultrasonography compared to MRI in defining tumor size, especially for the tumors >4 cm (22). Sozzi et al. showed a better diagnostic performance of ultrasonography compared to MRI and examination under anesthesia, especially for tumors >2 cm (14). In our opinion, combinatorial approaches with examination under anesthesia, ultrasonography, MRI, or PET/CT have important value in determining the tumor size. The presence of LVSI is considered as a risk factor for the PI, especially for the lymph node metastasis and local recurrence (23). In comparison to the DSI and negative PLN, the presence of LVSI may not be a similar important risk factor for the PI. In this study, the presence of LVSI had little effect on the risk for the patients in the PI with tumor size <2 cm. However, when tumor size is 2–4 cm, the effect of LVSI on the PI increases. A study by Coutant et al. reported that a combination of tumor size ≤2 cm and no LVSI may be feasible for selecting the patients who can benefit from a less radical surgery (13). Unlike other variables, the presence of LVSI can only be evaluated by using a conization sample. However, the detection of LVSI in biopsy or conization specimens lacks reliability and the negative predictive value of the detection of LVSI in the final pathological examination is low. In addition, the evaluation of LVSI by a qualitative method, as its presence or absence, presented obviously limited. A recent study reported that the patients with focal and diffuse LVSI had different prognosis and a semi-quantitative evaluation could better stratify the oncological outcomes (24). Considering the high risk of the PI for the larger tumors, it is very important to assess the status of LVSI before the decision of the less radical surgery.

It has been previously reported that PLN involvement can be a predictor of the PI. In this study, women with positive PLN were almost five times more likely to develop the PI compared to the negative PLN. However, knowledge of the lymph node status requires PLN dissection and it is not available before surgery. Several studies have investigated the contribution of the sentinel lymph node (SLN) biopsy to select the patients suitable for less radical surgery (25). In a prospective study, Strnad et al. reported that 133 negative PLN had no PI and 7 of 25 positive PLN had the PI (28%) (26). In this study, 120 of 3,574 patients with negative PLN and 321 of 959 patients with positive PLN had the PI. Therefore, the patients who are diagnosed with positive PLN by the preoperative imaging should be referred to the radiochemotherapy. For the patients with negative PLN in the preoperative imaging examination, intraoperative PLN status assessment has variable diagnostic value. Thus, before the less radical surgery, the patients with the early-stage CC require intraoperative PLN status assessment.

This study has multiple strengths. The cohort of this study is large with the availability of consistent clinical data. In this study, the data is recorded in detail, which allowed from incorporating more valuable predictors into the current model to improve the predictive accuracy. More importantly, most variables included in this score were significant in both the multivariate analysis and their clinical pertinence. This study is not without limitations. Because this study was conducted within a single academic health system, the data may not be generalizable to the population at large. In addition, the study is retrospective in nature and the bias in the process of the selection of the patient cannot be avoided. The independent external validation was not performed, which should be carried out to increase the reliability of the predictive model.

In conclusion, this study indicated that the combination of the preoperative features of the early-stage CC might help to select the patients with a less radical surgery and, hence, potentially contribute to a decrease in surgical morbidity. For the patients with tumor size ≤2 cm, no vaginal involvement, DSI <50%, and negative PLN were reported regardless of the presence of LVSI. Patients might benefit from the less radical surgery. For the patients with tumor size 2–4 cm, the absence of LVSI is necessary for the patients who would undergo the less radical surgery.

## Data Availability Statement

The original contributions presented in the study are included in the article/supplementary material, further inquiries can be directed to the corresponding author.

## Ethics Statement

The studies involving human participants were reviewed and approved by 2020-183. Written informed consent to participate in this study was provided by the participants' legal guardian/next of kin. Written informed consent was obtained from the individual(s) for the publication of any potentially identifiable images or data included in this article.

## Author Contributions

KH: protocol/project development. CL and SY: manuscript writing/editing, data analysis, and data collection or management. All authors contributed to the article and approved the submitted version.

## Funding

This study was supported by the Clinical Research Plan of SHDC (SHDC2020CR1045B) to KH, the Shanghai Municipal Health Commission (20194Y0085) to CL, and the Shanghai Rising Stars of Medical Talent Youth Development Program (SHWSRS2020087) to CL.

## Conflict of Interest

The authors declare that the research was conducted in the absence of any commercial or financial relationships that could be construed as a potential conflict of interest.

## Publisher's Note

All claims expressed in this article are solely those of the authors and do not necessarily represent those of their affiliated organizations, or those of the publisher, the editors and the reviewers. Any product that may be evaluated in this article, or claim that may be made by its manufacturer, is not guaranteed or endorsed by the publisher.
